# Chair-Side Direct Microscopy Procedure for Diagnosis of Oral Candidiasis in an Adolescent

**DOI:** 10.1155/2018/6561735

**Published:** 2018-04-29

**Authors:** Mathieu Lemaitre, Sarah Cousty, Mathieu Marty

**Affiliations:** ^1^Department of Biology, Toulouse Dental School, University of Toulouse III, Toulouse University Hospital, Toulouse, France; ^2^Department of Oral Surgery, Toulouse Dental School, University of Toulouse III, Toulouse University Hospital, Toulouse, France; ^3^Department of Pediatric Dentistry, Toulouse Dental School, University of Toulouse III, Toulouse University Hospital, Toulouse, France

## Abstract

Oral candidiasis is caused by fungi of the genus *Candida* and one of the most common opportunistic fungal infections of the human oral cavity. Given the clinical variability of this disease, microbiological techniques are often required for clinical confirmation, as well as establishing a differential diagnosis with other diseases. The aim of this brief technical report is to illustrate a simple chair-side method, which can provide immediate microscopic diagnosis of this disease. We present the case of a 14-year-old boy suffering from a denture-related erythematous stomatitis, diagnosed and followed-up with a simplified direct microscopy technique. It enables an accurate diagnosis with a noninvasive and painless sampling method, linked to laboratory results.

## 1. Introduction

Oral candidiasis is caused by fungi of the genus *Candida* and one of the most common opportunistic fungal infections of the human oral cavity. One hundred fifty species of this genus have been isolated in the oral cavity, 80% of which correspond to *Candida albicans* [1]. An oral candidiasis infection could either be acute or chronic, and classified as either pseudomembranous or erythematous. Oral candidiasis represents one of the most frequent mucosal diseases as all types of infection can be found [2]. An increased incidence of infection can be linked to various predisposing factors, such as prolonged antibiotic therapy, malnutrition, endocrine disorders, HIV infection, xerostomia, smoking, poor oral hygiene, and the use of prosthetic dentures [3]. Typically, a diagnosis of oral candidiasis is based on clinical symptoms [4] and usually straightforward in cases of acute pseudomembranous candidiasis, especially in infants. However, given the clinical variability of this disease, microbiological techniques are often required for clinical confirmation, as well as establishing a differential diagnosis with other diseases. In addition, cases characterized by resistance to antifungal drugs, particularly in chronic patients, may benefit from an additional microbiological test. Several methods are currently used to isolate and identify *Candida* species, including direct microscopy of smears, stains, cultures, and genetic methods (PCR) [5]. Also new identification methods are tested to provide rapid and accurate detection [6]. A recent literature review demonstrated the advantages of direct microscopy in the diagnosis of oral candidiasis in children and adolescents [7]. The aim of this brief technical report is to illustrate a simple chair-side method which can provide immediate microscopic diagnosis of this disease.

## 2. Case Report

**Figure 1:**
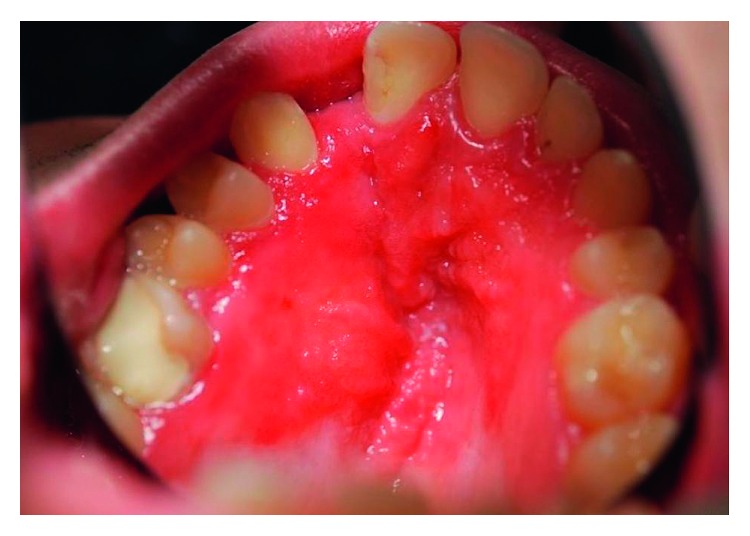
Erythematous, denture‐related candidiasis in a 14-year-old boy (photography taken in an intraoral mirror).

To illustrate this method, we present the case of a 14-year-old boy suffering from a denture-related erythematous stomatitis (Figure 1). Denture-related stomatitis is defined as an inflammatory process of the oral mucosa underlying a removable dental prosthesis. In young patients, a denture-related stomatitis could be due to a complication of a pediatric prosthetic denture or can occur during long-term orthodontic treatment with a removable material. This pathology has been described among both children and teenagers, and diagnosis methods and treatment have been proposed [8–11]. Here, a removable orthodontic appliance was worn for two years to compensate a dental agenesis.

A classical microscopical procedure typically involves removing a representative sample from the infected site (exfoliative cytology) which is transferred to a microscopic slide and treated with potassium hydroxide (KOH), Gram stain, or periodic acid-Schiff (PAS) stain. A sample collection was performed and sent to laboratory for cultivation and the result was positive for *Candida albicans*.

**Figure 2:**
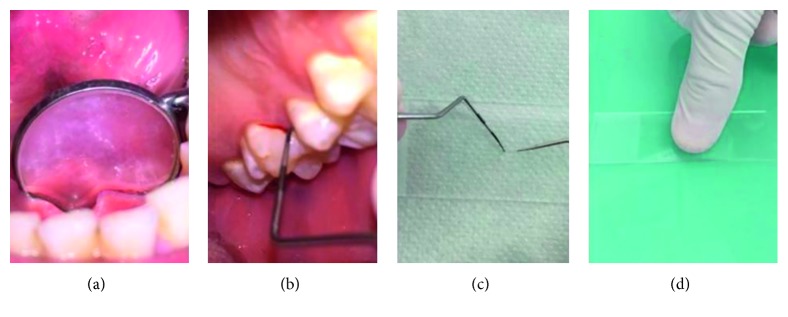
Step-by-step procedure for sampling.

**Figure 3:**
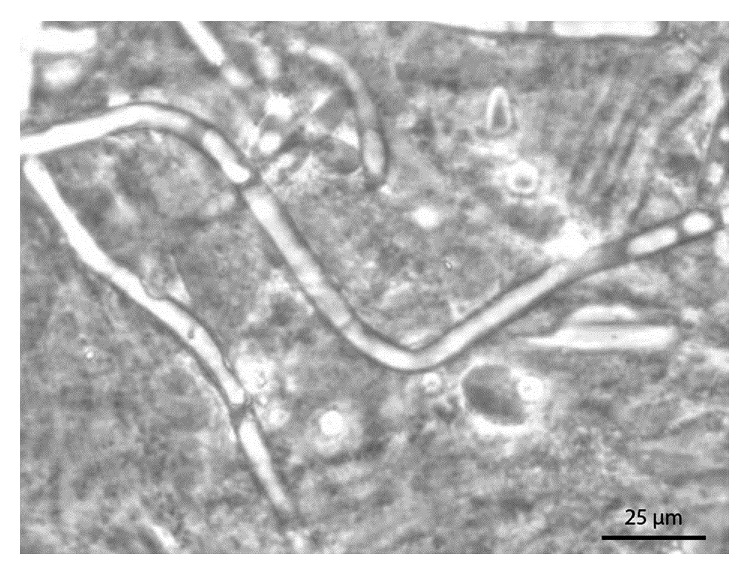
Candida hyphae (×1000 in patient's saliva).

**Figure 4:**
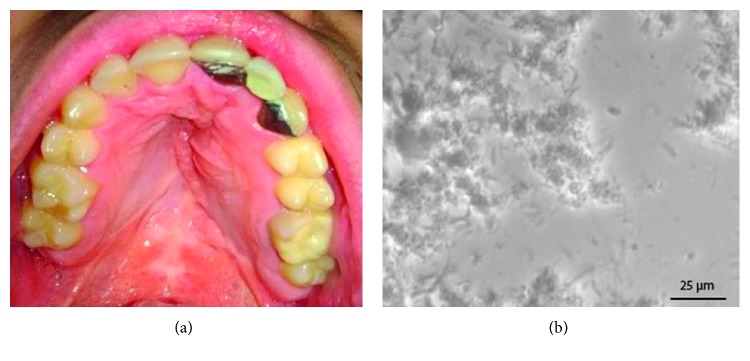
(a) Clinical improvement at the 2 years follow-up. (b) Normalization of oral flora after treatment (×1000 in patient's saliva).

Then we provided simplified, direct microscopy method as comparison. In this method, the patient's saliva was collected on the floor of the mouth. An intraoral mirror was placed horizontally beneath the tongue, in contact with mucosa. When saliva covers the mirror, it is removed from the mouth and laid on the slide (Figure 2(a)). The sample was subsequently collected with a sterile probe directly placed into the patient's saliva (Figure 2(c)), and a cover slip was mounted. An important point to note is that the sulcus area is the optimal site to collect the sample using this method (Figure 2(b)). The practitioner should press with his or her finger on the slide to spread the sample. The sample was then analyzed under a phase contrast optical microscope (Figure 2(d)). The most interesting magnification is ×1000 as it allows nonpathogenic yeast forms to be differentiated from opportunistic hyphal forms. In pathological conditions, several hyphae are visible on each screen, mixed with oral bacteria and cells (Figure 3). Importantly, this method is valuable for determining the efficacy of treatment. The treatment consisted in 3 weeks of local treatment using antifungal agent (amphotericin B), and the modification of the prosthetic appliance for a fixed one. An other laboratory analysis by cultivation was negative. At the two years follow-up, the same procedure showed a normalization of oral flora, with absence of hyphae (Figure 4(b)), linked to a clinical improvement (Figure 4(a)).

## 3. Conclusion

Direct microscopy is widely used in laboratories to diagnose candidiasis. It enables an accurate diagnosis with a noninvasive and painless sampling method, particularly well-suited to young patients. A classical procedure, which involves sample collection and shipment to a laboratory, takes more time and expense. Moreover, the method presented here does not require any fixation or sample treatment. Therefore, it is a cost-effective way to examine a patient's oral microbiota and determine any potential imbalance. Although direct microscopy is not as specific as culture, it appears to be a procedure of choice for a first-line diagnosis. Of course, a complete clinical examination remains absolutely necessary, as well as an extensive medical history, in order to study any associated general disease. Moreover, this approach requires experience in detecting yeast through microscopic observation which is not a transversal competence within clinicians. However the identification is not very difficult, and a simple, short-time formation may be sufficient. In this way, more practitioners will increasingly have access to this method; therefore, further awareness of this simple microscopic analysis will allow clinicians to make an immediate chair-side diagnosis. Furthermore, as it was the case for intraoral cameras, given the progress of microscopic devices, they can be integrated in the concept of person-centered care as an element of education and prevention to understand diagnoses and make informed treatment decisions [12]. Potential development of smartphone-based microscopes will provide easier access for clinicians to microscopy. Smartphone-based microscopes have already been in use to detect parasite [13] or obtain live-cells images [14]. This is a promising approach to perform accurate chair-side diagnosis in the future.
